# Public acceptability of population-level interventions to reduce alcohol consumption: A discrete choice experiment

**DOI:** 10.1016/j.socscimed.2014.05.010

**Published:** 2014-07

**Authors:** Rachel Pechey, Peter Burge, Emmanouil Mentzakis, Marc Suhrcke, Theresa M. Marteau

**Affiliations:** a Behaviour and Health Research Unit, Institute of Public Health, University of Cambridge, UK; b RAND Europe, Cambridge, UK; c Economics Department, School of Social Sciences, University of Southampton, Southampton, UK; d Health Economics Group, Norwich Medical School, University of East Anglia, Norwich, UK

**Keywords:** UK, Public acceptability, Alcohol, Health policy

## Abstract

Public acceptability influences policy action, but the most acceptable policies are not always the most effective. This discrete choice experiment provides a novel investigation of the acceptability of different interventions to reduce alcohol consumption and the effect of information on expected effectiveness, using a UK general population sample of 1202 adults. Policy options included high, medium and low intensity versions of: Minimum Unit Pricing (MUP) for alcohol; reducing numbers of alcohol retail outlets; and regulating alcohol advertising. Outcomes of interventions were predicted for: alcohol-related crimes; alcohol-related hospital admissions; and heavy drinkers. First, the models obtained were used to predict preferences if expected outcomes of interventions were not taken into account. In such models around half of participants or more were predicted to prefer the status quo over implementing outlet reductions or higher intensity MUP. Second, preferences were predicted when information on expected outcomes was considered, with most participants now choosing any given intervention over the status quo. Acceptability of MUP interventions increased by the greatest extent: from 43% to 63% preferring MUP of £1 to the status quo. Respondents' own drinking behaviour also influenced preferences, with around 90% of non-drinkers being predicted to choose all interventions over the status quo, and with more moderate than heavy drinkers favouring a given policy over the status quo. Importantly, the study findings suggest public acceptability of alcohol interventions is dependent on both the nature of the policy and its expected effectiveness. Policy-makers struggling to mobilise support for hitherto unpopular but promising policies should consider giving greater prominence to their expected outcomes.

## Highlights

• Novel UK-representative study predicting the acceptability of alcohol interventions.
• Acceptability varied by policy: regulating advertising was the most popular option.
• Acceptability increased for all policies with information on expected effectiveness.
• Information on expected effectiveness changed the order of preference for policies.

## 1 Introduction

Alcohol consumption and its consequences place a considerable burden on health services and policing, as well as imposing a myriad of less direct societal costs (Mohapatra et al., 2010; UK Government, 2007). Reviews of the evidence suggest three interventions that are likely to be particularly effective, which all happen to require government action: increasing alcohol prices, reducing the availability of outlets selling alcohol, and restricting the marketing of alcohol (Jackson et al., 2010; NICE, 2010; WHO Regional Office for Europe, 2009). While policy decisions may be informed by evidence on the effectiveness of potential policies and the costs of implementing them, public acceptability of interventions likely plays an even greater role. In addition, the accurate assessment of public acceptability is important as it can affect whether an intervention will work in practice.

A recent review of 200 studies of public acceptability of government interventions to change health-related behaviour, including a few studies on alcohol consumption, shows that public support is strongest for policies that are least intrusive, with greater support for educational campaigns than for taxation or restrictions on sales (Diepeveen et al., 2013). Respondents' own behaviour is also important, with those who do not engage in the targeted behaviour being more supportive of more intrusive intervention. For example, non- and ex-drinkers are more supportive of interventions such as higher taxes, bans on sales of alcohol in corner stores and bans on TV advertising (Giesbrecht et al., 2007; Holmila et al., 2009; Wilkinson et al., 2009). Acceptability also seems to be patterned by respondent demographic characteristics, with women and older people finding more intrusive interventions more acceptable (Bongers et al., 1998; Giesbrecht et al., 2007; Greenfield et al., 2007; Holmila et al., 2009; Wilkinson et al., 2009), while the impact of socioeconomic status is less clear.

The fact that existing studies are primarily based on standard opinion polls and surveys, along with the observed lack of public acceptability of increasing price to reduce alcohol consumption has led to calls for research to better understand this (WHO Regional Office for Europe, 2009). A recent focus group study revealed scepticism about the effectiveness of minimum unit pricing with resultant limited support for this policy intervention (Lonsdale et al., 2012). Providing information about the effectiveness of policies can increase their acceptability, as shown in an experimental study concerning the use of financial incentives for smoking cessation and weight loss (Promberger et al., 2012). It is unknown, however, whether a similar effect will be seen for alcohol policies when the interventions may have a direct impact upon the public by, for example, making alcohol less accessible or less affordable.

The aim of the current study is to examine public acceptability of three interventions to reduce alcohol consumption and to examine the extent to which acceptability is sensitive to information about the effectiveness of the interventions. We explore both the size of any effect as well as the domain in which it is reported (crime vs. health). The influence of respondent characteristics, particularly drinking habits, gender and socio-economic status, is also explored.

## 2 Methods

### 2.1 Participants

1202 adults resident in England participated in the discrete choice experiment (DCE) during face-to-face interviews conducted in their homes in November and December 2013. Recruitment was carried out by a market research company at 30 sampling points across England, with quotas set on age, gender and occupational group (UK Registrar General's classification: Higher Managerial and Professional (groups A&B); White Collar and Skilled Manual (groups C1&C2); and Semi-skilled and Unskilled Manual (groups D&E)). The sample was broadly representative of the English population (Table 1), including, importantly, in terms of participants' drinking habits (i.e. non-drinkers, moderate drinkers or heavy drinkers) (Health and Social Care Information Centre, 2012). Ethical approval for the study was obtained from the University of Cambridge Psychology Research Ethics Committee (Ref: Pre.2012.61).

### 2.2 Attributes and levels

Participants were presented with a range of policy options alongside different policy outcomes with regard to three domains (alcohol-related crimes, alcohol-related hospital admissions, heavy drinkers). The key attributes were: (1) the type of intervention (minimum unit pricing, reduction in outlet density, regulation of advertising); (2) the intensity of the intervention (low, medium, high); and, (3) the magnitude of impact in each of the three outcome domains (9 levels, representing the reductions in each domain in a community of 100,000 people over a year) (see Supplementary Materials for details).

### 2.3 Policy options: intervention type and intensity

The three levels of intensity for each intervention type were selected such that they encompassed at least one option that was currently being discussed in the UK policy context, as well as looking at how increasing the intensity (and potential effectiveness) of these would impact on acceptability: setting a minimum unit price for alcohol of (a) 40p, (b) 70p or (c) £1; reducing the number of outlets that sell alcohol by (a) 10%, (b) 20% or (c) 40%; and regulating advertising of alcohol via (a) self-regulation, (b) a partial ban or (c) a complete ban. Advertising regulations were described as: Self-regulation: ‘Work with industry to make sure they are following the current rules (For example, the government would look at ways to make sure that adverts for alcohol are not shown during programmes for children or teenagers)’; Partial ban: ‘Part ban on alcohol adverts (Adverts for alcohol would be banned from TV and in cinemas)’; Complete ban: ‘Complete ban on adverts for alcohol (All adverts for alcohol would be banned)’.

### 2.4 Policy outcomes: domain and magnitude of impact

The impacts on each of the three domains were estimated using and extrapolating from the modelling of the Sheffield Alcohol Research Group (Purshouse et al., 2009, 2010) (see Supplementary Materials for details, Table S11 for estimates used). As these estimates are based on several assumptions (see Supplementary Materials), in what follows we refer to those estimates as the best available outcome estimates associated with the interventions. Alcohol-related crimes and hospital admissions were defined as in the Sheffield models (i.e. crimes: 20 types including theft, criminal damage, assault and causing death by dangerous driving; hospital admissions: both those wholly or partly attributable to alcohol, and due to both acute and chronic conditions). Heavy drinkers were defined as those drinking more than UK government guidelines (22 units or more per week for men, 15 units or more per week for women).

The best available outcome estimates allow us to examine the acceptability of policies taking into account their predicted impact. However, in order to avoid confounding between intervention effects and outcome effects, the outcomes presented to participants in the study were not constrained to the most likely estimates for each policy, but were drawn independently from any of the nine estimates associated with that outcome.

### 2.5 Experimental design

**Fig. 1:**
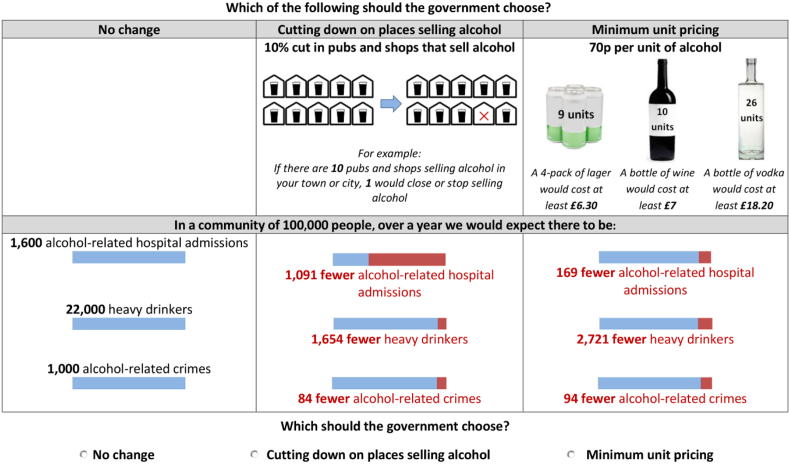
Example choice set.

Three-way choices (choice sets) were presented to participants: two options describing types of intervention, alongside an option representing no change to the status quo (see Fig. 1 for an example choice set). The ordering of the two active policy interventions in the scenarios was varied using a random draw. Each type of intervention was presented at one of the three levels of intensity, with the outcomes presented for all three domains for each option (each outcome at one of the nine levels of magnitude). Participants were asked to ‘imagine the effects shown would be the real effects for the options’. For each choice, participants decided which of the options (either of the two presented policy interventions or ‘no change’) the government should choose to implement.

Such discrete choice experiments are based on the idea that the acceptability of a policy is characterised by certain selected attributes and that participants' own acceptance of any policy depends on these attributes. Although there are some limitations inherent in such a design (i.e. the preferences examined are hypothetical, and the attributes chosen cannot reflect the full spectrum of options), this does allow us to examine the influence of said attributes on participants' (albeit hypothetical) preferences, and has been widely used in health-related studies (Ryan, 2004). See Supplementary Materials for more on the DCE methodology used.

The DCE used 81 different choice sets (determined using an orthogonal main effects fractional factorial design), divided into nine blocks of nine. Each participant completed one block. A blocking approach was used to specify the subsets of scenarios to present to each respondent in order to minimise the correlation between each individual attribute and the block selected; this ensured that each respondent was presented a range of scenarios with differences in both policy options and outcomes.

### 2.6 Analysis

Heteroskedastic conditional logit models (bootstrapped to correct for any specification error introduced through using multiple responses from each respondent) were used to model participants' preferences between the different policy options. See Supplementary Materials for model details.

In the results section we present the predicted probabilities that different policy options would be chosen if the sample of respondents were to be offered the choice of that policy or no intervention. Two models are used: the first is estimated with common coefficients across all respondents to provide average values for the sample, the second takes into account statistically significant differences between subgroups. The models were then used to predict preferences in two situations: first, how participants would respond if they had no information regarding the effectiveness of each policy, and second, how participants would respond if they were informed of the best available outcome estimates for each policy.

## 3 Results

**Fig. 2:**
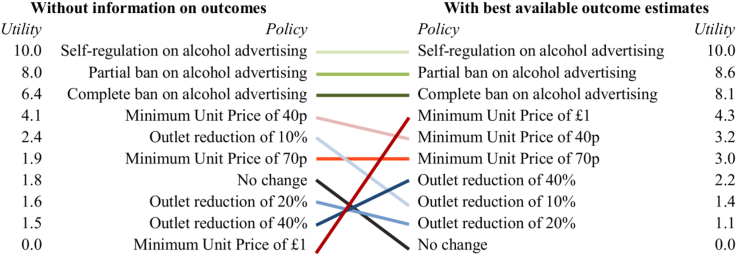
Utility of each policy option, modelled with and without information on outcomes (normalised to scale: 10 = best, 0 = worst).

Fig. 2 shows the predicted relative utility of (ranked preferences for) each of the policy options when taking into account the impact of withholding or providing the best available estimates of each of the outcomes. If outcome estimates are not taken into account, participants would prefer minimum unit pricing (MUP) of 40p and any regulation of advertising over the status quo (all *p* < 0.001). Four policies are equally preferable to the status quo (MUP of 70p, and all three levels of outlet reduction), while MUP of £1 is less preferable than the status quo (*p* < 0.001).

If outcome estimates are taken into account, the rankings of the different intervention types change (given that some interventions were expected to be more effective than others), with all policy options preferred over the status quo. The ranking of higher intensity MUP improves in particular.

**Fig. 3:**
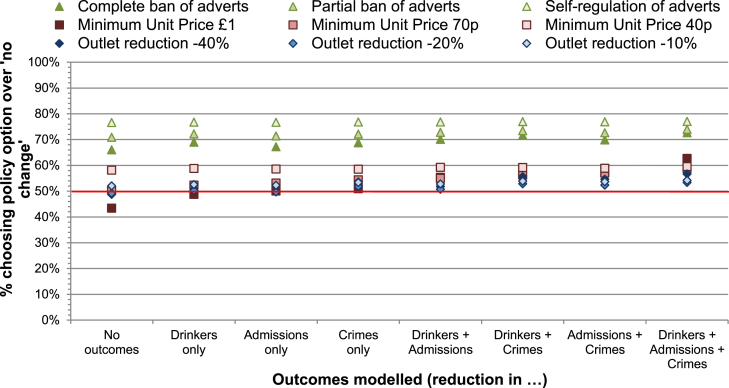
Proportion predicted to choose each policy option over status quo, by outcomes modelled.

The percentage of the sample predicted to choose to implement a given policy option rather than choose to maintain the status quo is shown in Fig. 3. Separate predictions were made for each intervention and for the various combinations of information on outcomes that may be available to individuals when making their choices. For example, if participants were choosing between a partial ban on adverts or making 'no change' to the status quo in the absence of any information on expected outcomes, approximately 70% of participants would be predicted to favour the partial ban. Indeed, for the case where participants have no information on outcomes (i.e. effectiveness of intervention), 58% are predicted to choose MUP of 40p over the status quo, and between 66 and 77% would prefer advertising regulations to the status quo; the other policies would be chosen over the status quo by around 50% of the sample, with the exception of MUP of £1, preferred by only 43% of respondents.

By contrast, in cases where information on all three outcome estimates is available to participants, reductions in outlet density are predicted to be the least preferred options, with 53–57% choosing these over the status quo, compared to 59–63% for MUP interventions. Advertising regulations remain the most acceptable, with 73–77% predicted to choose these over the status quo.

While the type of intervention clearly impacts on its relative acceptability (with a difference of 34 percentage points predicted between the most and least acceptable policies in the case where no information is available regarding outcome estimates), presenting outcome estimates in all three domains would reduce this difference by 10 percentage points, and alter the relative ranking of policies. This is particularly evident for MUP interventions, the acceptability of which changes markedly in absolute and relative terms between these two cases: the proportion of the sample choosing MUP of £1 over the status quo increases by 20 percentage points (from no outcome estimates known to all three known), and its ranking improves from last to fourth place, even higher than that of the less intensive MUP options.

### 3.1 Subgroup analysis

**Fig. 4:**
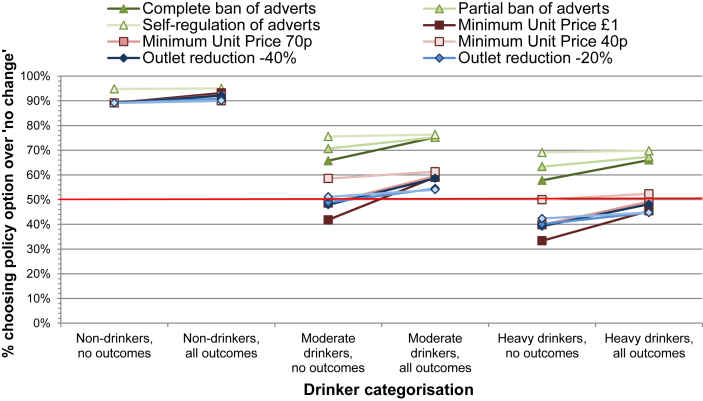
Proportion predicted to choose each policy option over status quo, by drinker categorisation.

Taking into account statistically significant differences between subgroups, the most notable differences were by drinker status (see Fig. 4), with non-drinkers placing a significantly higher value on all policies (with the exception of self-regulation of advertising) relative to the status quo (all *p* < 0.05). In both cases where no outcome estimates are available and where all three outcome estimates are available, 89%–95% of non-drinkers would choose a given policy over the status quo. By contrast, drinkers discriminated more between the different intervention types, with individual policies being preferred to the status quo by between 42 and 76% of moderate drinkers and between 33 and 69% of heavy drinkers (in predictions for the case without available outcome estimates). Whilst non-drinkers are seen to be relatively insensitive to information on policy outcomes, the predictions show that in contrast drinkers are influenced by such information (in predictions including all three outcome estimates 59–76% of moderate drinkers would choose a given policy over the status quo, compared to 45–70% for heavy drinkers). In general, a lower proportion of heavier than moderate drinkers would prefer a given policy to the status quo, with an approximately 10 percentage point difference for most policies. Differences in model scale, however, show that non-drinkers had more unexplained error in their choices (i.e. were harder to predict) relative to moderate drinkers (parameter = 0.50, *p* < 0.01), whereas hazardous and harmful drinkers were more certain in their choices compared to moderate drinkers (parameter = 1.35, *p* < 0.01).

Systematic analyses were conducted by demographic characteristics (see supplementary materials Table S14). Within the moderate drinkers group (comprising 64% of the study sample and 62% of the English population (Health and Social Care Information Centre, 2012)), males were more likely to choose to maintain the status quo (*p* < 0.05), whereas infrequent drinkers (drinking less than once a week) and those in the three highest occupational groups A, B & C1 were less likely to favour no change (both *p* < 0.001). There were no other differences found by gender or occupational group, and no differences found by age, highest educational qualification or working status.

In terms of outcomes, reductions in the numbers of alcohol-related crimes showed a linear relationship with acceptability, while hospital admissions were linearly valued up to a reduction of 213 admissions, but with additional reductions beyond this point providing no extra value. In general, reductions in the number of heavy drinkers were not greatly valued by participants, with the exception to this pattern being amongst the female moderate drinkers (*p* < 0.001).

## 4 Discussion

The relative preferences for the three intervention types in the discrete choice models (minimum unit pricing, outlet reduction and regulating advertising) suggest that policies regarding regulation of marketing would be most acceptable to participants. Interestingly, however, whether or not information on the best available outcome estimates of these policy options is available influences the absolute and relative acceptability of the interventions, with acceptability increasing as more outcomes are taken into consideration. Indeed, in predictions where information on all three outcome estimates is available, a majority of participants are predicted to prefer any given policy to the status quo, and the gap in acceptability between regulating advertising and other policy options reduces compared to the case where no information on outcome estimates is available. In particular, MUP (which has greater expected effectiveness with regard to the investigated outcomes compared to the other intervention types) has similar levels of acceptability to outlet reductions in the predictions where no information is available on outcome estimates, but is preferred to outlet reduction policies in the case where information is available on all outcome estimates. In absolute terms, for MUP of £1 (the least popular option when no information is available on outcome estimates), an additional fifth of the sample would prefer this policy over the status quo should they have information on the outcome estimates, making this the preferred policy option after marketing regulations in this case. While there were significant differences in preferences by drinker status, with the models suggesting that heavier drinkers found all policies less acceptable, there were no marked differences by other respondent characteristics.

The varying levels of acceptability by type of intervention reflect findings from surveys conducted in Canada and the US that showed more support for bans on TV advertising of alcohol than for changes in price and restrictions on sales (albeit in the forms of increased taxes on alcoholic beverages and limits on the hours stores can sell alcohol) (Giesbrecht et al., 2007; Greenfield et al., 2007). Evidence for acceptability varying by effectiveness of intervention has similarly been shown in studies looking at acceptability of financial incentives (Promberger et al., 2012), and suggested in focus groups as a factor limiting public support with regard to alcohol policies (Lonsdale et al., 2012), but, to our knowledge, has not been empirically demonstrated in this context before, nor quantified in this way. The findings from the current study reflect one of very few experimental studies looking at acceptability, offering a more rigorous assessment of acceptability (by confronting respondents with trade-offs between different policy options) compared to the existing acceptability literature which mainly relies on asking respondents to provide unrestricted, hypothetical answers in opinion polls.

The findings with regard to respondent characteristics also largely tie in with previous research (Diepeveen et al., 2013): primarily consisting of surveys suggesting that individuals' behaviour is a key factor in predicting policy acceptability, with heavier drinkers being less supportive (Giesbrecht et al., 2007; Holmila et al., 2009; Wilkinson et al., 2009). Moreover, a small number of surveys have looked at attitudes to alcohol control policies by socioeconomic status (SES), but found weak, if any, patterns of support. Together with the current study's findings, this suggests that attitudes to alcohol control are largely not mediated by SES, but perhaps these mixed findings reflect the complex relationships between SES and drinking behaviour (Bloomfield et al., 2006; Erskine et al., 2010).

The findings from this large sample, broadly representative of the English population, provide novel experimental evidence of the relative acceptability of different policies across different demographic groups. A further advantage of the approach taken in this study was the assessment of topical interventions, combined with using estimates of likely outcomes (albeit based on several assumptions), to allow a timely and policy relevant appraisal of their public acceptability. There are two main limitations to the current study, however: firstly, it focused in a somewhat stylized manner only on the impact of positive outcomes, whereas a policy discussion is likely to involve multiple and competing messages; and secondly, participants were asked to ‘imagine the effects shown would be the real effects for the options’, while it is unlikely that the public place absolute trust in figures presented by parties with a stake in policy choices. Another possible concern is that participants were told that heavy drinkers comprised those ‘*who drink more alcohol than the government advises*’ but participants may have assumed different thresholds for heavy drinking if they were unaware of government recommendations. Further investigation of these influences would add nuance to the study findings in terms of the relative influence of likely policy outcomes on public acceptability.

The results suggest the most favoured policies in this study (restrictions on advertising) also had the lowest estimates of effectiveness (in terms of the investigated outcomes). However, presentation of (beneficial) outcomes when conveying a policy may help boost its public acceptability; an implication that policymakers struggling in mobilising support for ex ante less acceptable but more effective interventions (e.g. MUP) could build on. Moreover, the patterning of support by drinking status provides an impetus for government action, given that as alcohol consumption worsens, so too will the likely acceptability of any interventions to target relevant behaviours.

Future research into the acceptability of policies to change behaviour should build on these results to place them more in the context of real world policy deliberations, by taking into account how the message is framed (e.g., targeting particular groups of the population) and improving understanding of the role that varying degrees of uncertainty around the outcome estimates might play.

## Figures and Tables

**Table 1:** Participant characteristics (*N* = 1202).

Variable	*n* (%)
*Gender*	
Male	555 (46.2)
Female	647 (53.8)
*Age*	
18–34	332 (27.6)
35–54	454 (37.8)
55+	416 (34.6)
*Occupational group*	
A&B (Higher managerial and professional)	383 (31.9)
C1&C2 (White collar and skilled manual)	527 (43.8)
D&E (Semi-skilled and unskilled manual)	292 (24.3)
*Drinking habits*^a^	
Non-drinker	158 (13.1)
Moderate drinker	763 (63.5)
Heavy drinker	281 (23.4)
*Highest educational qualification*	
No formal qualification	199 (16.6)
GCSE or equivalent	475 (39.5)
A-level or equivalent	196 (16.3)
Degree or higher	259 (21.5)
Other/still studying/don't know	73 (6.1)
*Working status*	
Working full-time	543 (45.2)
Working part-time	184 (15.3)
Not working	472 (39.3)
Don't know/refused	3 (0.2)

a Heavy drinkers: reported drinking more than UK government guidelines of 21 units a week for men and 14 for women over the past week; Moderate drinkers: reported drinking within guidelines over the past week; Non-drinkers: reported drinking no alcohol over the past 12 months.
