# Supplementary material for: Public acceptability of population-level interventions to reduce alcohol consumption: A discrete choice experiment
Source: Soc Sci Med. 2014 Jul;113(100):104–9. doi: 10.1016/j.socscimed.2014.05.010 (PMC4065329; doi:10.1016/j.socscimed.2014.05.010)
Supplement: Supplementary file 1 [file mmc1.docx]

**Supplementary materials: Public acceptability of population-level interventions to reduce alcohol consumption: a discrete choice experiment**

**Contents**

[**A. Producing estimates of outcomes** 2](#_Toc386785908)

[Table S1. Estimates of the effects of minimum unit pricing on heavy drinkers. 3](#_Toc386785909)

[Table S2. Estimates of the effects of minimum unit pricing on alcohol-related hospital admissions and crimes. 3](#_Toc386785910)

[Table S3. Studies considered in Purshouse et al (2009) to estimate the effect of a 10% reduction in alcohol outlets. 4](#_Toc386785911)

[Table S4. The effect of MUP on overall consumption vs. hazardous and harmful consumption 4](#_Toc386785912)

[Table S5. Estimates of the distribution of mean reduction in consumption across hazardous and harmful drinkers for outlet density reductions. 5](#_Toc386785913)

[Table S6. Estimates of absolute reductions in the numbers of hazardous and harmful drinkers for outlet density reductions. 6](#_Toc386785914)

[Table S7. Estimates of the effects of outlet density reductions on alcohol-related crimes and hospital admissions. 6](#_Toc386785915)

[Table S8. Estimates of the distribution of mean reduction in consumption across hazardous and harmful drinkers for advertising regulations. 7](#_Toc386785916)

[Table S9. Estimates of absolute reductions in the numbers of hazardous and harmful drinkers for advertising regulations. 7](#_Toc386785917)

[Table S10. Estimates of the effects of advertising regulations on alcohol-related hospital admissions and crimes. 8](#_Toc386785918)

[Table S11. Estimated reductions per 100,000 people per year in each domain used in the study 8](#_Toc386785919)

[**B: Design of the choice sets** 9](#_Toc386785920)

[Table S12. The attributes and levels used in the study. 9](#_Toc386785921)

[Table S13. Use of a random draw to determine the order of presentation of the policy options. 10](#_Toc386785922)

[**C. Estimation of the discrete choice models** 11](#_Toc386785923)

[Figure S1. Contribution to utility of intervention by alcohol-related hospital admissions. 13](#_Toc386785924)

[**D. Results of analyses by demographic characteristics** 15](#_Toc386785925)

[Table S14. Revised choice model taking into account differences by sub-groups 15](#_Toc386785926)

[References 16](#_Toc386785927)

## A. **Producing estimates of outcomes**

We have estimated the effects for England, using as our key references two publications produced by the Sheffield group:

*Purshouse et al (*[*2010*](#_ENREF_7)*). Estimated effect of alcohol pricing policies on health and health economic outcomes in England: an epidemiological model. Lancet 2010; 375: 1355–64.*

*Purshouse et al (*[*2009*](#_ENREF_6)*). Modelling to assess the effectiveness and cost-effectiveness of public health related strategies and interventions to reduce alcohol attributable harm in England using the Sheffield Alcohol Policy Model version 2.0. Report to the NICE Public Health Programme Development Group*

Those studies cover all three types of policies (minimum unit pricing, outlet density reduction, advertising regulation), but they do not cover all three levels of each of the three policies. In particular, there is no estimate of a £1 minimum unit price (MUP), and there is no estimate in the literature for 20% or 40% reductions in outlet density, nor for self-regulation or partial bans on advertising. Plus in some cases, Sheffield uses other metrics than we require here: Purshouse et al ([Purshouse et al. 2009](#_ENREF_6); [Purshouse et al. 2010](#_ENREF_7)) present the outcomes in terms of reduction in consumption rather than reduced prevalence of types of drinkers. Hence the need to add further assumptions. In what follows, we describe how we derived the relevant values for each of the policies.

***Estimating the effects of MUP:***

Purshouse et al ([2010](#_ENREF_7)) provide estimates for the “full effect” (that would be reached at 10 years post implementation) resulting from a wide range of pricing policies on alcohol consumption (and other outcomes). Our estimates for heavy drinkers were derived as shown in Table S1, and estimates for hospital admissions and crimes were derived as shown in Table S2:

Table S1. Estimates of the effects of minimum unit pricing on heavy drinkers.

|  | *MUP* | *Hazardous drinker* | *Harmful drinker* | *Total (hazardous+harmful)* |
| --- | --- | --- | --- | --- |
| Baseline harm |  | 8,300,000 | 2,900,000 | 11,200,000 |
| Percentage reduction in mean consumption per week (units) | 40p | 1.8% | 4.5% |  |
|  | 70p | 18.5% | 24.1% |  |
|  | £1* | 51.5% | 55.3% |  |
| Absolute reductions ** | 40p | 149,400 | 130,500 | 279,900 |
|  | 70p | 489,700 | 298,700 | 788,400 |
|  | £1* | 1,535,500 | 698,900 | 2,234,400 |

* Purshouse et al ([Purshouse et al. 2009](#_ENREF_6); [Purshouse et al. 2010](#_ENREF_7)) did not model the effect of MUP=£1. We have estimated the associated outcomes according to a polynomial function, which resulted from plotting several MUPs against the estimated % reductions in Purshouse et al ([Purshouse et al. 2010](#_ENREF_7)). The regression line gave a fit of R^2^=0.99 and thus allowed us to extrapolate beyond the MUPs in the article.

** Assuming that the percentage reduction in mean consumption translates into the same percentage reduction in number of hazardous/harmful drinkers.

Table S2. Estimates of the effects of minimum unit pricing on alcohol-related hospital admissions and crimes.

|  | *MUP* | *Reduction per year* | *Reduction per year in a community of 100,000* |
| --- | --- | --- | --- |
| Hospital admissions | 1 | *561,498* | *1091* |
|  | 0.7 | 226,400 | 440 |
|  | 0.5 | 92,200 | 179 |
|  | 0.4 | 37,800 | 73 |
|  | 0.3 | 8,900 | 17 |
|  | 0.2 | 900 | 2 |
| Crimes | 1 | 236,550 | 460 |
|  | 0.7 | 123,700 | 240 |
|  | 0.6 | 83,200 | 162 |
|  | 0.5 | 42,500 | 83 |
|  | 0.4 | 10,100 | 20 |

Note: the available estimates from Purshouse et al ([2010](#_ENREF_7)) related to the MUPs from 0.2-0.7. We extrapolated the effect of the MUP=£1 from the polynomial function relating MUP to reductions in hospital admissions for the existing estimates and from the linear function between MUP and reduction in crimes. Both functions had a near perfect fit (R^2^>0.99).

***Estimating the effects of outlet density reductions:***

The only available estimate from Purshouse et al ([2009](#_ENREF_6)) related to a 10% reduction in outlets: we took the median estimate from the studies considered (Table S3):

### **Table S3**. Studies considered in Purshouse et al ([2009](#_ENREF_6)) to estimate the effect of a 10% reduction in alcohol outlets.

|  | *Effect on units of consumption (%)* | *Effect on hospital admissions (in 1,000s)* | *Effect on crime (in 1,000s)* |
| --- | --- | --- | --- |
| Blake & Nied ([1997](#_ENREF_1)), model 3 | -2.3 | -24.6 | -60.9 |
| Gruenewald et al ([1993](#_ENREF_3)) | -3.7 | -43.5 | -83.9 |
| Hoadley et al ([1984](#_ENREF_4)) | -0.3 | -3.2 | 6.2 |
| Schonlau et al ([2008](#_ENREF_8)) | -1 | -11.9 | -22.8 |
| Xie et al ([2000](#_ENREF_9)) | -1.9 | -22.6 | -43.4 |
| **Median estimate** | -1.9 | -22.6 | -43.4 |

Purshouse et al ([Purshouse et al. 2009](#_ENREF_6); [Purshouse et al. 2010](#_ENREF_7)) present the effect of outlet density reduction on mean reduction in alcohol units consumed, but not on hazardous and harmful drinking specifically, which was the focus of our study. In the absence of any further benchmarks, the assumption we adopted in transforming the relevant outcomes was that the distribution of the response (across different types of drinkers) to changes in outlet density is the same as that to MUP policies. Unfortunately, however, that distribution differs between the pricing policies, e.g. between MUP=40p and MUP=70p (see Table S4).

Table S4. The effect of MUP on overall consumption vs. hazardous and harmful consumption (according to Purshouse et al ([2009](#_ENREF_6))).

| *MUP* | *Changes in overall mean consumption* | *Changes in hazardous consumption* | *Changes in harmful consumption* | *Changes in hazardous consumption as proportion of overall change* | *Changes in harmful consumption as proportion of overall change* |
| --- | --- | --- | --- | --- | --- |
| 40p | 2.40% | 1.80% | 4.50% | 75% | 187.5% |
| 70p | 17.50% | 18.50% | 24.10% | 105.7% | 138% |

Table S5 provides our estimates on how the mean reduction in consumption is distributed across hazardous and harmful drinking. For a fairly “low” intensity policy (assumed to be 10-20% outlet reduction), we assumed the same distribution (i.e. 75% of the mean goes to hazardous, 187.5% to harmful drinking) as in the fairly low intensity MUP interventions (assumed to be a 40p MUP). For higher intensity policies (assumed to be >20% outlet reduction) we assumed the same distribution as that for the high intensity MUP intervention (MUP=70p). We then converted the percentages in Table S5 into absolute reductions (Table S6) (which we then converted into equivalent figures for a community of 100,000 - not shown here).

We also had to make assumptions about the effects across all relevant outcomes resulting from outlet reductions higher than 10% (the only estimate available from Purshouse et al ([2009](#_ENREF_6))). We assumed a linear effect, to err on the cautious side. The alternative would have been a non-linear effect, as is the case for pricing policies, where the marginal effect increases with the level of MUP.

Table S5. Estimates of the distribution of mean reduction in consumption across hazardous and harmful drinkers for outlet density reductions.

| *Outlet density reduction* | *Mean alcohol consumption reduction (%)* | *Reduction in hazardous drinkers (%)* | *Reduction in harmful drinkers (%)* | *Assumptions* |
| --- | --- | --- | --- | --- |
| 10% | 1.9 | 1.4 | 3.6 | 75 and 187.5% distribution |
| 20% | 3.8 | 2.9 | 7.1 | 75 and 187.5% distribution |
| 30% | 5.7 | 4.3 | 10.7 | 75 and 187.5% distribution |
| 40% | 7.6 | 8.0 | 10.5 | 105.7 and 138% distribution |
| 50% | 9.5 | 10.0 | 13.1 | 105.7 and 138% distribution |

Table S6. Estimates of absolute reductions in the numbers of hazardous and harmful drinkers for outlet density reductions.

| *Outlet density reduction* | *Reduction in hazardous drinkers* | *Reduction in harmful drinkers* | *Total* |
| --- | --- | --- | --- |
| 10% | 118,275 | 103,313 | 221,588 |
| 20% | 236,550 | 206,625 | 443,175 |
| 30% | 354,825 | 309,938 | 664,763 |
| 40% | 666,846 | 303,522 | 970,368 |
| 50% | 833,557 | 379,403 | 1,212,960 |
| Baseline number of drinkers* | 8,300,000 | 2,900,000 | 11,200,000 |

* Purshouse et al ([2010](#_ENREF_7))

The effect on crimes and on hospital admissions “only” had to rely on the assumption of the linear effect, i.e. doubling the reduction resulted in double the reduced number of crimes/admissions (Table S7).

Table S7. Estimates of the effects of outlet density reductions on alcohol-related crimes and hospital admissions.

| *Outlet density reduction* | *Absolute reduction in crimes* | *Absolute reduction in hospital admissions* |
| --- | --- | --- |
| 10% | 43,400 | 22,600 |
| 20% | 86,800 | 45,200 |
| 30% | 130,200 | 67,800 |
| 40% | 173,600 | 90,400 |
| 50% | 217,000 | 113,000 |

***Estimating the effects of advertising regulation:***

The only directly related, available estimate in the Sheffield modelling results comes from a total ban effect. Purshouse et al ([2009](#_ENREF_6)) emphasise that there are only two underlying studies, one of which finds a counter-intuitive alcohol consumption increasing effect and the other finding a rather notable consumption reducing effect. For our estimates we take the mean estimate of the two existing estimates Purshouse et al ([2009](#_ENREF_6)) refer to (i.e. 12.5% reduction in mean consumption). Again we need to assume how the change in mean consumption is distributed between harmful and hazardous drinking. Those assumptions are given below in Table S8 and again emulate the distribution that was estimated for the MUP interventions.

- For the partial ban effect, in the absence of any available estimates, we assumed a 50% lower effect than in the case of the total ban.
- For the self-regulation option, in the absence of any available estimates, we assume 10% of the estimated total ban effect.

These percentages were then transformed into absolute reductions for the population of England (Table S9).

Table S8. Estimates of the distribution of mean reduction in consumption across hazardous and harmful drinkers for advertising regulations.

| *Advertising regulation* | *Change in mean consumption* | | | *Change in hazardous drinkers (%)* | *Change in harmful drinkers (%)* | *Assumptions as to how to distribute the mean effect to hazardous and harmful consumption* |
| --- | --- | --- | --- | --- | --- | --- |
|  | High estimate | Low estimate | Mean |  |  |  |
| Self-regulation | NA | NA | -1.3% | -0.94% | -2.34% | 75 and 187% distribution |
| Partial ban | NA | NA | -6.3% | -4.69% | -11.72% | 75 and 187% distribution |
| Complete ban | -29.90% | 4.90% | -12.50% | -13.2% | -17.1% | 105.7 and 138% distribution |

Table S9. Estimates of absolute reductions in the numbers of hazardous and harmful drinkers for advertising regulations.

| *Advertising regulation* | *Hazardous drinkers* | *Harmful drinkers* | *Total* |
| --- | --- | --- | --- |
| Baseline harm* | 8,300,000 | 2,900,000 | 11,200,000 |
| Self-regulation | -77,813 | -67,969 | -145,781 |
| Partial ban | -389,063 | -339,844 | -728,906 |
| Complete ban | -1,096,638 | -496,625 | -1,593,263 |

* Purshouse et al ([2010](#_ENREF_7))

To estimate the effect on admissions and crime we proceed similarly and obtain the following effects, in terms of absolute numbers for England (Table S10).

Table S10. Estimates of the effects of advertising regulations on alcohol-related hospital admissions and crimes.

|  | *Advertising regulation* | *High estimate* | *Low estimate* | *Mean* |
| --- | --- | --- | --- | --- |
| Hospital admissions | Self-regulation |  |  | -10,955 |
|  | Partial ban |  |  | -54,775 |
|  | Complete ban | -279,900 | 60,800 | -109,550 |
| Crimes | Self-regulation |  |  | -9,680 |
|  | Partial ban |  |  | -48,400 |
|  | Complete ban | -238,200 | 44,600 | -96,800 |

**Final Estimates:** Table S11 shows the final estimates of reductions per 100,000 people per year in each domain used in the study.

Table S11. Estimated reductions per 100,000 people per year in each domain used in the study *(intervention and intensity on which each estimate is based in parentheses*)*

| **Outcome level** | **Alcohol-related crimes** | | **Alcohol-related hospital admissions** | | **Heavy drinkers** | |
| --- | --- | --- | --- | --- | --- | --- |
| 1 | 19 | *(RA: Self-regulation)* | 21 | *(RA: Self-regulation)* | 272 | *(RA: Self-regulation)* |
| 2 | 20 | *(MUP: 40p)* | 44 | *(OR: 10%)* | 414 | *(OR: 10%)* |
| 3 | 84 | *(OR: 10%)* | 73 | *(MUP: 40p)* | 544 | *(MUP: 40p)* |
| 4 | 94 | *(RA: Partial ban)* | 87 | *(OR: 20%)* | 827 | *(OR: 20%)* |
| 5 | 161 | *(OR: 20%)* | 106 | *(RA: Partial ban)* | 1360 | *(RA: Partial ban)* |
| 6 | 188 | *(RA: Complete ban)* | 169 | *(OR: 40%)* | 1532 | *(MUP: 70p)* |
| 7 | 240 | *(MUP: 70p)* | 213 | *(RA: Complete ban)* | 1654 | *(OR: 40%)* |
| 8 | 296 | *(OR: 40%)* | 440 | *(MUP: 70p)* | 2721 | *(RA: Complete ban)* |
| 9 | 460 | *(MUP: £1)* | 1091 | *(MUP: £1)* | 4342 | *(MUP: £1)* |
| Status quo | 1000 |  | 1600 |  | 22000 |  |

*MUP: Minimum unit pricing; OR: Outlet reduction; RA: Regulating advertising*

** Estimates for each domain were drawn independently from any of the nine possible levels of each outcome, and were not constrained to the most likely estimates*

## B: **Design of the choice sets**

The first attribute in Table S12 was used to eliminate one of the three policy interventions from the choice set. In addition, a random draw was used to specify which order to present the remaining two policy interventions (and their associated outcomes) in order to control for any potential ordering bias (see Table S13).

Table S12. The attributes and levels used in the study.

| ***Attributes*** | ***Number of levels*** | ***Levels*** |
| --- | --- | --- |
| Which intervention type does not appear in the choice set | 3 | Minimum unit pricing; Outlet reduction; Regulating advertising |
| Intensity level of Minimum unit pricing | 3 | 40p; 70p; £1.00 |
| Reduction in number of crimes for Minimum unit pricing | 9 | 19; 20; 84; 94; 161; 188; 240; 296; 460 |
| Reduction in number of hospital admissions for Minimum unit pricing | 9 | 21; 44; 73; 87; 106; 169; 213; 440; 1091 |
| Reduction in number of heavy drinkers for Minimum unit pricing | 9 | 272; 414; 544; 827; 1360; 1532; 1654; 2721; 4342 |
| Intensity level of Outlet reduction | 3 | 10%; 20%; 40% |
| Reduction in number of crimes for Outlet reduction | 9 | 19; 20; 84; 94; 161; 188; 240; 296; 460 |
| Reduction in number of hospital admissions for Outlet reduction | 9 | 21; 44; 73; 87; 106; 169; 213; 440; 1091 |
| Reduction in number of heavy drinkers for Outlet reduction | 9 | 272; 414; 544; 827; 1360; 1532; 1654; 2721; 4342 |
| Intensity level of Regulating advertising | 3 | Self-regulation; partial ban; complete ban |
| Reduction in number of crimes for Regulating advertising | 9 | 19; 20; 84; 94; 161; 188; 240; 296; 460 |
| Reduction in number of hospital admissions for Regulating advertising | 9 | 21; 44; 73; 87; 106; 169; 213; 440; 1091 |
| Reduction in number of heavy drinkers for Regulating advertising | 9 | 272; 414; 544; 827; 1360; 1532; 1654; 2721; 4342 |

Table S13. Use of a random draw to determine the order of presentation of the policy options.

| ***Which intervention type does not appear in the choice set*** | ***Random draw*** | ***Resulting design*** | | |
| --- | --- | --- | --- | --- |
|  |  | ***Alternative A*** | ***Alternative B*** | ***Alternative C*** |
| Minimum unit pricing | 0 | No change | Outlet density reduction | Regulating advertising |
| Outlet density reduction | 0 | No change | Minimum unit pricing | Regulating advertising |
| Regulating advertising | 0 | No change | Minimum unit pricing | Outlet density reduction |
| Minimum unit pricing | 1 | No change | Regulating advertising | Outlet density reduction |
| Outlet density reduction | 1 | No change | Regulating advertising | Minimum unit pricing |
| Regulating advertising | 1 | No change | Outlet density reduction | Minimum unit pricing |

## C. **Estimation of the discrete choice models**

The discrete choice models developed are based on random utility theory and aim to replicate the stated choices made during the study. The models are based on the principle that each respondent acts to maximise their utility, i.e. chooses the policy alternative which they believe is the best of those on offer. Each choice alternative, *i*, in a discrete choice model is specified with a utility function for respondent, *n*, such that:

$U_{ni} = V_{ni } + \varepsilon_{ni}$

V_ni_ is the systematic or measurable utility that the individual *n* receives from adopting choice *i*. In addition to these observed components, the utility functions contain error terms ε_ni_ that account for the unobserved components of utility.

McFadden ([1974](#_ENREF_5)) showed that working from the assumption that the individual is a utility maximiser when considering the alternatives, *j*, available to them, i.e.

$$P_{ni} = \text{Prob} (V_{ni} + \varepsilon_{ni} > V_{nj }+ \varepsilon_{nj} \forall j\neq i)$$

and that the error terms ε_ni_  are assumed to be independent and identically distributed (iid) with a Gumbel distribution, that it is possible to derive a succinct closed form expression for the logit choice probability:

$\lambda$is the scale parameter and is inversely proportional to the standard deviation. Under the iid assumption $\lambda$can be fixed (i.e. normalize standard deviation and variance). Relaxing this assumption and making scale a function of covariates we are able to characterize how the noise (i.e. unobserved component) in individuals' choices varies by individual characteristics. This formulation is known as the heteroskedastic conditional logit ([DeShazo and Fermo 2002](#_ENREF_2)). As there is an assumption of independence between observations[[1]](#footnote-1), the likelihood function is given by the product of the model probabilities that each individual chooses the option that they are actually observed to select; and the models are fit through maximum likelihood.

***Development of the specification of the utility functions:***

The models have been developed by exploring a range of different specifications of the utility equations used to represent the factors influencing the choice of each alternative.

Firstly, the extent to which outcomes were valued linearly was tested. This was undertaken by defining the levels on each of the outcome attributes as a series of dummy terms within the model and plotting these estimates, along with their confidence intervals, to reveal the extent to which changes within the outcome level appeared to be valued linearly or otherwise. For those cases where we believed that the valuations could be non-linear, piecewise linear specifications were tested to allow a point of inflexion. The preferred formulation was determined on the basis of tests of model fit, using the likelihood ratio test to compare alternative specifications to a linear specification.

The one outcome attribute for which a piecewise linear formulation was found to be preferred was the number of hospital admissions. For this we found that the parsimonious model providing the best model fit was one which included a point of inflexion at 213 per 100,000 alcohol-related hospital admissions; with the function showing gains in utility up until this point and then a plateau from thereon, suggesting no further value being placed on higher levels of outcome (see Figure S1).


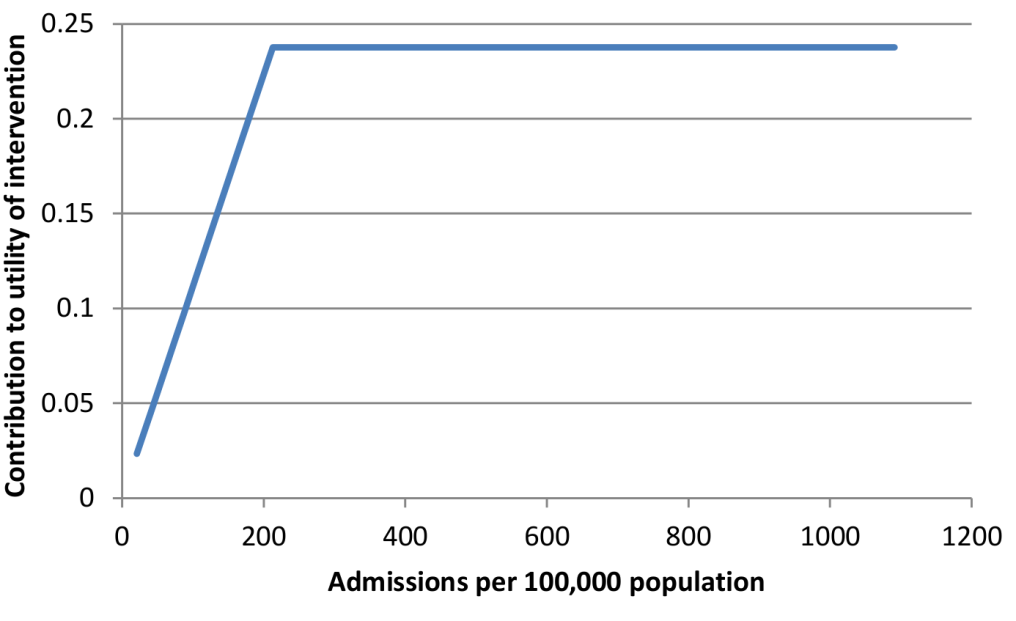


Figure S1. Contribution to utility of intervention by alcohol-related hospital admissions.

Secondly, we systematically compared model forecasts with observed choices to test whether respondent characteristics affected how participants valued certain policies or outcomes. For cases where it appeared that the model did not accurately reproduce the observed choice behaviour, we then introduced additional covariates into the model to test whether the previous model had been underspecified and whether the new covariates were statistically significant. These tests were undertaken in a systematic manner to review the potential influence of each of the key demographic characteristics available within the dataset. From these tests we identified that the respondent’s existing level of drinking played a major role in the valuation of the underlying attractiveness of each of the policies. We further observed statistical differences within the moderate drinkers group, with differences in propensity to choose the “no change” option by gender, social grade, and level of weekly alcohol consumption, and differences in the value placed on reducing the number of heavy drinkers according to gender.

Finally, correlations between alternatives were examined, to look for different substitution patterns. A nested logit formulation was tested which allowed different substitution rates between the two policy options and the “no change” option. However, the likelihood ratio test showed that this model was not found to provide a statistically better fit to the data.

## D. Results of analyses by demographic characteristics

### Table S14. Revised choice model taking into account differences by sub-groups

| **Coefficient** | **Estimate** | **95% CI ^a^** |
| --- | --- | --- |
|  |  |  |
| **Policies ^b^** |  |  |
| Minimum Unit Price of 70p, drinkers | -0.4125 | -0.5247 to -0.3003 |
| Minimum Unit Price of £1, drinkers | -0.6928 | -0.8330 to -0.5526 |
| Availability -10%, drinkers | -0.3080 | -0.4370 to -0.1791 |
| Availability -20%, drinkers | -0.4013 | -0.5386 to -0.2641 |
| Availability -40%, drinkers | -0.4381 | -0.5411 to -0.3350 |
| Self-regulation on alcohol advertising, drinkers & non-drinkers | 0.8043 | 0.6662 to 0.9424 |
| Partial ban on alcohol advertising, drinkers | 0.5480 | 0.4460 to 0.6500 |
| Full ban on alcohol advertising, drinkers | 0.3151 | 0.1941 to 0.4361 |
| No change, Non-drinkers | -2.1023 | -4.5165 to 0.3119 |
| No change, Moderate drinkers – male | 0.2449 | 0.0292 to 0.4606 |
| No change, Moderate drinkers – social grade A, B, C1 | -0.5017 | -0.7392 to -0.2643 |
| No change, Moderate drinkers – drink less than once a week | -0.4273 | -0.6827 to -0.1719 |
|  |  |  |
| **Outcomes (Reduction in alcohol related …)** |  |  |
| Crimes (per crime per 100,000 people) | 0.0006 | 0.3800 to 0.8032 |
| Hospital admissions (per admission per 100,000 people), Up to 213 admissions ^c^ | 0.0011 | 1.0322 to 2.5382 |
| Hospital admissions (per admission per 100,000 people), Beyond 213 admissions ^c^ | -0.0011 | -1.0322 to -2.5382 |
| Heavy drinkers (per drinker per 100,000 people), Moderate drinkers - female ^d^ | 0.0001 | 0.9398 to 3.0329 |
|  |  |  |
| **Model scale (error)** |  |  |
| Non-drinkers | 0.4973 | 0.1943 to 0.8003 |
| Moderate drinkers | 1.0000 | n/a |
| Hazardous and harmful drinkers | 1.3468 | 1.0886 to 1.6050 |
|  |  |  |
| **Summary statistics** |  |  |
| Observations | 10818 |  |
| Final Log Likelihood | -10605.5 |  |
| Rho²(0) | 0.108 |  |
| Rho²(c) | 0.072 |  |

^a^ Confidence intervals estimated from bootstrapped model

^b^ Base is “no change” and MUP 40p for drinkers, and all policies other than self-regulation for non-drinkers, specified as such as differences between these were not found to be statistically significant

^c^ Admissions is added a piece-wise linear function, with no statistically significant value placed on improvements beyond 213 admissions per 100,000 people

^d^ All other categories were absorbed into the base as they were not-significantly different

## References

Blake, D., and A. Nied. 1997. The demand for alcohol in the United Kingdom. *Applied Economics* 29: 1655-72.

DeShazo, J.R., and G. Fermo. 2002. Designing Choice Sets for Stated Preference Methods: The Effects of Complexity on Choice Consistency. *Journal of Environmental Economics and Management* 44: 123-43.

Gruenewald, P.J., W.R. Ponicki, and H.D. Holder. 1993. The Relationship of Outlet Densities to Alcohol Consumption: A Time Series Cross-Sectional Analysis. *Alcoholism: Clinical and Experimental Research* 17: 38-47.

Hoadley, J.F., B.C. Fuchs, and H.D. Holder. 1984. The effect of alcohol beverage restrictions on consumption: a 25-year longitudinal analysis. *American Journal of Drug & Alcohol Abuse* 10: 375-401.

McFadden, D. 1974. The measurement of urban travel demand. *Journal of public economics* 3: 303-28.

Purshouse, R.C., A. Brennan, N. Latimer, Y. Meng, R. Rafia, R. Jackson, and P.S. Meier. 2009.*Modelling to assess the effectiveness and cost-effectiveness of public health related strategies and intervention to reduce alcohol attributable harm in England using the Sheffield Alcohol Policy Model version 2.0*. University of Sheffield. Report to the NICE Public Health Programme Development Group.

Purshouse, R.C., P.S. Meier, A. Brennan, K.B. Taylor, and R. Rafia. 2010. Estimated effect of alcohol pricing policies on health and health economic outcomes in England: an epidemiological model. *The Lancet* 375: 1355-64.

Schonlau, M., R. Scribner, T.A. Farley, K.P. Theall, R.N. Bluthenthal, M. Scott, and D.A. Cohen. 2008. Alcohol outlet density and alcohol consumption in Los Angeles county and southern Louisiana. *Geospatial health* 3: 91-101.

Xie, X., R.E. Mann, and R. Smart. 2000. The direct and indirect relationships between alcohol prevention measures and alcoholic liver cirrhosis mortality. *Journal of Studies on Alcohol and Drugs* 61: 499.

1. An ex-post correction for the violation of this assumption was made through bootstrapping the models, sampling individuals rather than choice observations, to correct for any specification error introduced through the consideration of multiple responses from each respondent. [↑](#footnote-ref-1)
